# Severe Anorectal Mpox With Newly Diagnosed HIV and Syphilis: A Diagnostic and Management Challenge

**DOI:** 10.7759/cureus.98708

**Published:** 2025-12-08

**Authors:** Juan R Santos-Rivera, Regina McPherson, Guillermo Izquierdo-Pretel

**Affiliations:** 1 Internal Medicine, Ponce Health Sciences University, Ponce, PRI; 2 Internal Medicine, Florida International University, Herbert Wertheim College of Medicine, Miami, USA; 3 Hospital Medicine, Jackson Memorial Hospital, Miami, USA

**Keywords:** genital lesions, hiv, monkeypox, orthopoxvirus, stis, syphillis

## Abstract

Mpox has re-emerged globally in non-endemic regions, challenging clinicians with diverse mucocutaneous manifestations that frequently mimic other sexually transmitted infections (STIs). Early genital and anorectal lesions may resemble herpes simplex virus or syphilis, often resulting in diagnostic error and treatment delay. Lesion distribution frequently corresponds to the route of transmission, further complicating clinical recognition. We describe a 25-year-old male who presented with diffuse pustular and vesicular eruptions, including extensive anorectal and genital involvement, confirmed as Mpox by polymerase chain reaction (PCR) testing. Additional evaluation revealed previously undiagnosed HIV infection with marked immunosuppression and latent syphilis. This case underscores the diagnostic complexity and therapeutic urgency of Mpox in patients with concurrent STIs and highlights the necessity of comprehensive sexual-health screening, early antiviral initiation, and multidisciplinary management during ongoing outbreaks.

## Introduction

In 2022, Mpox re-emerged as a significant global health concern, with thousands of cases reported across non-endemic regions, including the United States [1,2]. Mpox is caused by a double-stranded DNA virus belonging to the Orthopoxvirus genus and is primarily transmitted through direct contact with infected body fluids, lesions, or contaminated materials such as clothing and bedding [2,3]. Additional transmission routes include close face-to-face interactions, skin-to-skin contact, and sexual activity, particularly among individuals with multiple partners. Recent outbreaks have shown a predominance of lesions in the anorectal and genital regions, complicating clinical recognition and raising concerns about sexual transmission pathways [3,4].

The clinical course of Mpox typically progresses through four cutaneous stages - macular, papular, vesicular, and pustular - culminating in crusting and resolution over two to four weeks [5]. These manifestations frequently overlap with those of sexually transmitted infections (STIs) such as herpes simplex virus and syphilis, making accurate diagnosis particularly difficult in high-risk populations [5,6]. Immunocompromised individuals, including those living with HIV, may present with more severe disease or atypical features, further blurring clinical distinctions. The anogenital distribution of lesions seen in recent outbreaks has highlighted the need for comprehensive sexual health evaluation in suspected cases [4,6].

Although real-time PCR remains the diagnostic gold standard, Mpox presentations may vary substantially, especially in the setting of coinfection [7]. This case report describes a 25-year-old male with Mpox, newly diagnosed HIV infection, and latent syphilis. The case underscores the diagnostic complexity and management challenges posed by overlapping infectious diseases and reinforces the importance of multidisciplinary care. Understanding how Mpox interacts with concurrent infections is essential for optimizing patient outcomes and informing effective public health responses [8,9]. While Mpox presentations involving genital or perianal lesions have been reported, cases with severe anorectal disease occurring alongside newly diagnosed HIV and latent syphilis are exceedingly rare. This report highlights the diagnostic and therapeutic challenges of managing concurrent viral and sexually transmitted infections with overlapping clinical features.

## Case presentation

**Figure 1:**
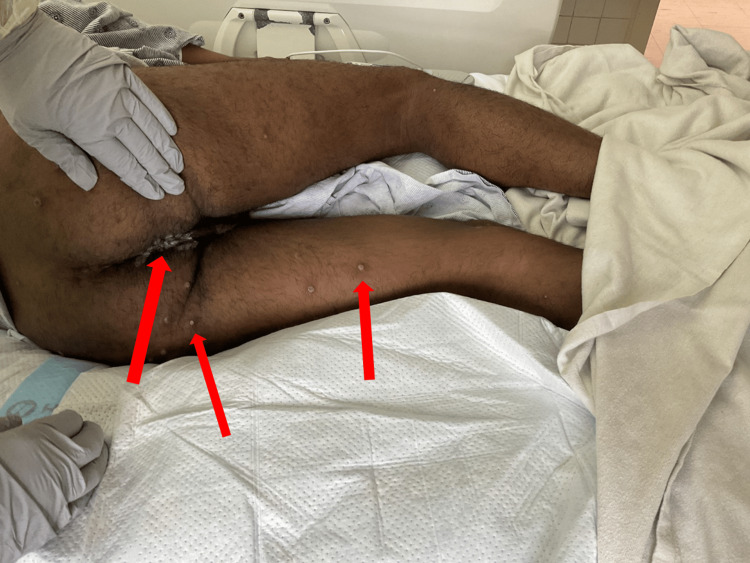
Perianal and rectal lesions showing pustular nodules with surrounding inflammation. Arrows indicate pustular nodules and areas of surrounding inflammation.

In June 2024, a 25-year-old male with no known past medical history presented to a tertiary hospital in Florida with rectal bleeding, rectal pain, and newly developed anal lesions of two days’ duration. He was initially diagnosed with herpes simplex virus and discharged with acyclovir. However, five days later, he returned to the emergency department due to worsening symptoms, including the development of diffuse vesicular lesions across his body and painful verrucous lesions surrounding the anus (Figure 1).

The patient reported unprotected anal intercourse two weeks prior with a male partner who disclosed being on pre-exposure prophylaxis (PrEP). He denied any history of prior STIs and had no recent travel, animal exposure, or systemic symptoms prior to the initial presentation.

**Figure 2:**
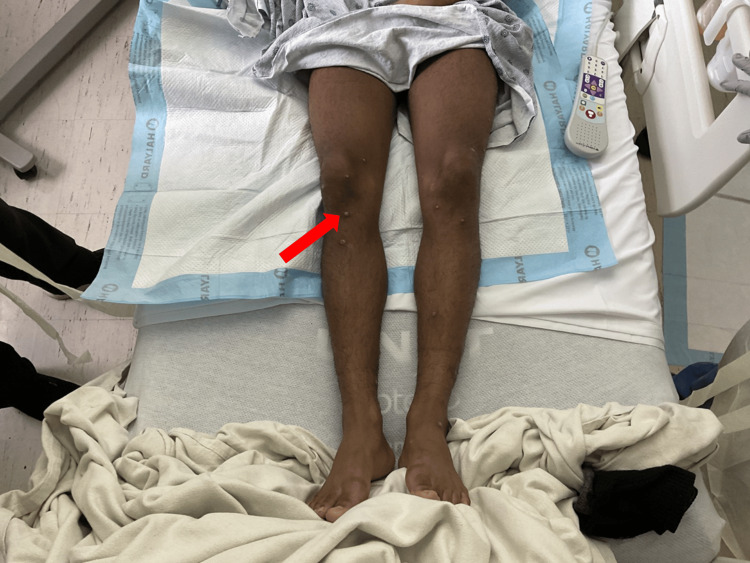
Papular lesions on the lower limbs, notably around the knees and shins. Arrow indicates the papular lesion around the knee.

**Figure 3:**
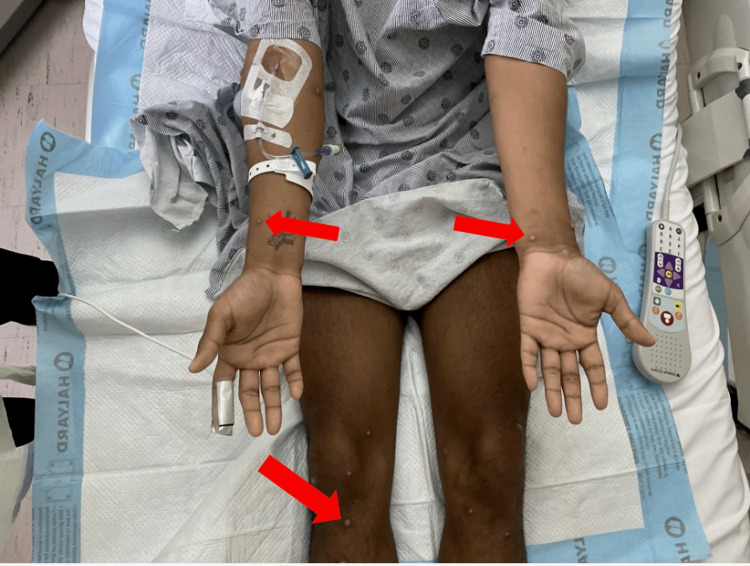
Scattered papular lesions on the extremities, with notable sparing of palms and fingers. Arrows indicate scattered papular lesions on the extremities.

**Figure 4:**
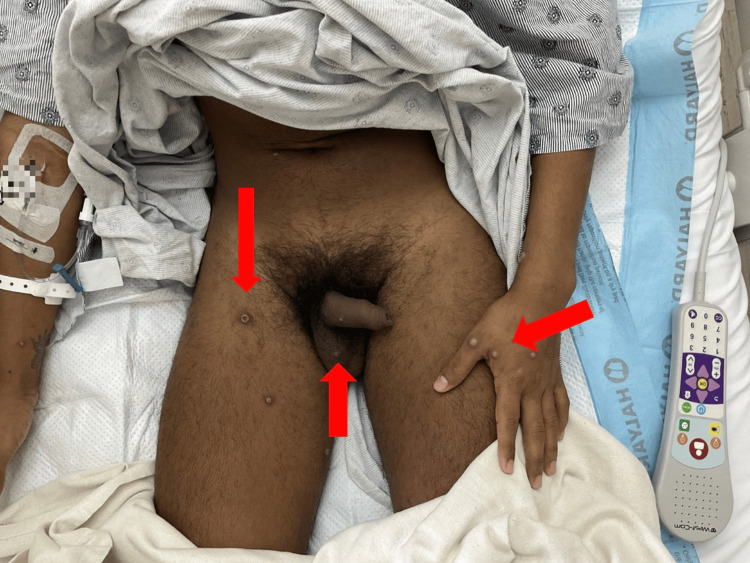
Pustular and vesicular lesions on the genital region, including the penile shaft and inner thighs. Arrows indicate pustular and vesicular lesions.

**Figure 5:**
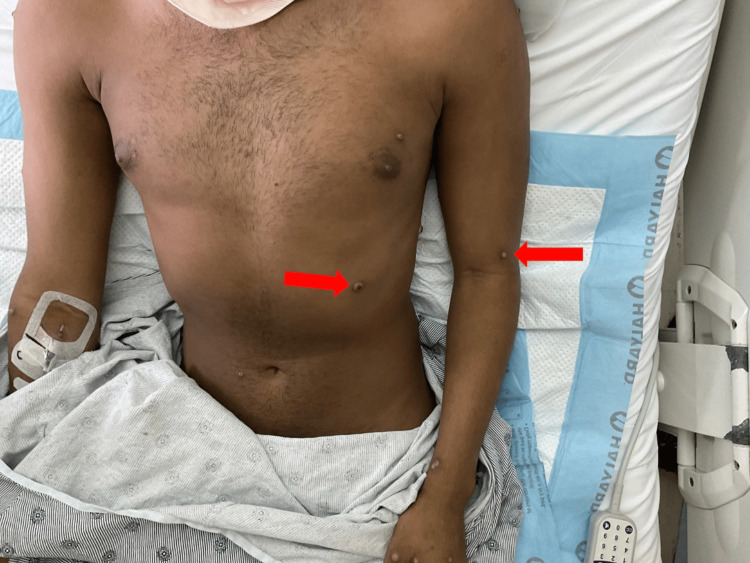
Annular pustular lesions on the abdomen and medial forearm. Arrows indicate the annular pustular lesions on the abdomen and medial forearm.

**Figure 6:**
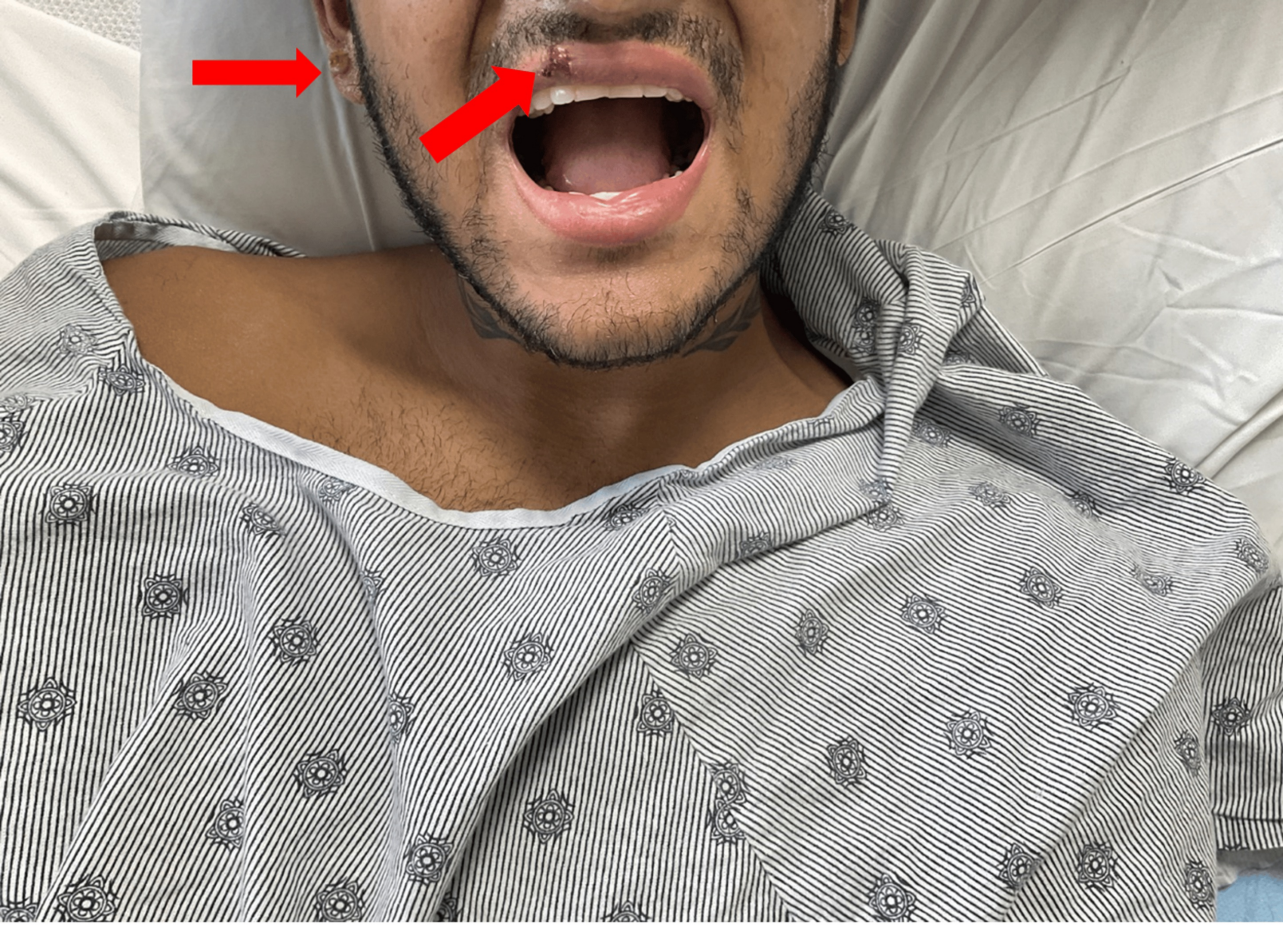
Ulcerated lesion on the upper lip mucosa and a crusted pustular lesion on the left auricular region. Arrows indicate lesions on the upper lip and the left auricular region.

On clinical examination, his vital signs were within normal limits. Physical findings included left inguinal and right axillary lymphadenopathy. Skin lesions were observed across the extremities, lips, face, torso, genitals, and perianal region (Figures 2-5). These lesions were annular, raised, and pustular in appearance, measuring approximately 1 cm in diameter. Notably, the perianal area exhibited extensive verrucous changes and ulcerations (Figure 6).

Laboratory investigations demonstrated abnormalities in white blood cell count and revealed several infectious diagnoses, including Mpox, HIV, and syphilis. The patient tested positive for Mpox via polymerase chain reaction PCR. HIV testing revealed a positive result with a low CD4 count and elevated viral load, and syphilis serology showed a reactive rapid plasma reagin (RPR) titer. A summary of key laboratory findings is provided in Table 1.

**Table 1:** Summary of Pertinent Laboratory Findings ¹RPR = Rapid Plasma Reagin, a non-treponemal serologic test for syphilis ²PCR = Polymerase Chain Reaction, confirmatory test for monkeypox virus DNA

Laboratory Parameter	Result	Reference Range	Interpretation
White blood cell count	11.5 × 10⁹/L	4.0–10.0 × 10⁹/L	Elevated
HIV viral load	3,210 copies/mL	Undetectable	Detectable
CD4⁺ T-cell count	354 cells/μL	500–1,600 cells/μL	Decreased
CD4/CD8 ratio	0.15	0.9–3.6	Decreased
CD4⁺ percentage	0.082	30–60%	Decreased
Rapid plasma reagin (RPR)¹	0.044444	Nonreactive	Reactive (Latent Syphilis)
Mpox PCR²	Positive	Negative	Positive

The patient was admitted under the care of the infectious disease service. Contact and droplet precautions were instituted. Supportive measures included pain control and skin care. Due to the severity of the facial, genital, and perianal lesions, antiviral therapy with tecovirimat was initiated in accordance with Centers for Disease Control and Prevention (CDC) recommendations. Antiretroviral therapy was started using bictegravir, emtricitabine, and tenofovir alafenamide (Biktarvy®), and intramuscular penicillin G benzathine was administered weekly for latent syphilis.

Public health reporting and follow-up 

The patient’s case was reported to the Florida Department of Health and enrolled in its Mpox program for research and surveillance. Follow-up appointments were scheduled with ID specialists and primary care for continued management of Mpox, HIV, and syphilis. He was discharged in stable condition with instructions on medication adherence, infection prevention strategies, and follow-up care.

## Discussion

Mpox lesions are typically firm or rubbery, well-circumscribed, and deep-seated, often progressing to central umbilication. Recent outbreaks have predominantly involved genital, anorectal, and oral lesions, either isolated or accompanied by lesions across other body regions [2,3]. Evidence suggests a correlation between lesion distribution and transmission routes, with anorectal and genital lesions frequently observed in men who have sex with men (MSM) [4,5]. This presentation often mimics other STIs, complicating diagnosis. Mpox lesions characteristically progress through four stages - macular, papular, vesicular, and pustular - before crusting and resolution over two to four weeks [2,6]. The clinical overlap with other STIs underscores the importance of including Mpox in the differential diagnosis, particularly in patients with coinfections that may obscure both presentation and management [4,7].

There is currently no universally approved curative therapy for Mpox. Management primarily consists of supportive care and preventive measures, including isolation and lesion coverage until crusting is complete and viral shedding has ceased [8]. For severe or high-risk cases, including those with extensive mucocutaneous involvement or immunocompromising conditions, the antiviral Tecovirimat is recommended under expanded access protocols. Tecovirimat acts by inhibiting the viral envelope protein VP37, disrupting viral maturation and release [9]. In this case, its use was warranted due to severe involvement of facial, genital, and anorectal regions. Timely access to antiviral therapy is essential, particularly for patients with coinfections or delayed healing [9,10].

This case highlights the importance of maintaining a broad differential diagnosis in patients presenting with anorectal or genital lesions. The patient was initially misdiagnosed with herpes simplex virus infection, reflecting the clinical overlap in genital lesions during the early stage of Mpox. However, as the disease progressed, the later-stage lesions became more characteristic of Mpox, with deep-seated, well-circumscribed pustules and umbilication. The lesion distribution and the patient’s MSM epidemiologic context further supported Mpox as the correct diagnosis. Initial screening should include testing for HIV, hepatitis C, syphilis, HSV-1, HSV-2, and Mpox to ensure timely identification of all potential coinfections [5,11]. A multidisciplinary approach involving dermatology, infectious disease, and public health services is essential for accurate diagnosis, effective isolation, and prevention of transmission. While PCR remains the gold standard for confirmation, its limited accessibility in some settings underscores the need for heightened clinical suspicion and improved diagnostic infrastructure [6,12].

Similar to previously reported cases, our patient’s presentation occurred in the setting of immunocompromise, which likely contributed to the extent and severity of mucocutaneous disease. Prior studies have described Mpox in HIV-positive individuals with additional comorbidities such as tuberculosis or secondary infections, showing ocular, genital, or disseminated lesions and prolonged recovery despite supportive therapy [13-15]. Furthermore, early Caribbean reports described patients with painful genital and perianal ulcers in MSM, underscoring the diagnostic overlap between Mpox and sexually transmitted infections in this population [16]. In contrast, our case demonstrated extensive verrucous anorectal involvement and mucosal destruction in a newly diagnosed HIV-positive patient, representing an atypical and aggressive phenotype. Collectively, these findings suggest that immunosuppression and mucosal tropism may predispose to severe, atypical Mpox manifestations, reinforcing the need for early recognition and multidisciplinary management.

Finally, this case illustrates the diagnostic challenges posed by Mpox when coinfections such as HIV and syphilis are present. Overlapping clinical features may lead to misdiagnosis or delayed treatment, particularly in patients with multiple STIs or immunosuppression. By including Mpox in the differential diagnosis for genital or anorectal lesions - especially in MSM or patients with a history of high-risk sexual activity - clinicians can improve diagnostic accuracy and facilitate timely, appropriate management. This case also reinforces the importance of robust public health preparedness, expanded antiviral access, and comprehensive multidisciplinary care in managing coinfections during ongoing outbreaks [4,5,9].

## Conclusions

This case highlights the need for heightened diagnostic vigilance when assessing genital or anorectal lesions, particularly among individuals with high-risk sexual behavior or potential coinfections. The patient’s initial misdiagnosis illustrates how early Mpox lesions can mimic other sexually transmitted infections, emphasizing the value of comprehensive STI screening and confirmatory molecular diagnostics. Lesion distribution patterns may offer important insight into both transmission route and disease severity. Recognition of disseminated or atypical presentations in immunocompromised hosts is critical for guiding timely antiviral therapy and multidisciplinary coordination. Ultimately, improved clinician awareness and sustained public-health readiness are essential to enhance diagnostic accuracy and optimize outcomes in current and future Mpox outbreaks.
